# Consented Universal Newborn Screening for Congenital Cytomegalovirus Infection Using Dried Blood Spot and Dried Saliva Spot Specimens: Implementation and Relative Programmatic Accuracy

**DOI:** 10.3390/ijns12040085

**Published:** 2026-10-12

**Authors:** Emeril Santander, Emily Reuvers, Melanie Lacaria, Stacey Hume, Zeinab Moazin, Lauren Gallagher, Danielle Durie, Alan Mears, Kristin D. Kernohan, Soren Gantt, Ari Bitnun, Jessica K. E. Dunn, Pranesh Chakraborty, Jason Brophy, Neal Sondheimer

**Affiliations:** 1 Newborn Screening Ontario, Children’s Hospital of Eastern Ontario, Ottawa, ON K1H 8M8, Canadaereuvers@cheo.on.ca (E.R.); shume@cheo.on.ca (S.H.); lgallagher@cheo.on.ca (L.G.); ddurie@cheo.on.ca (D.D.);; 2 Children’s Hospital of Eastern Ontario Research Institute, Children’s Hospital of Eastern Ontario, Ottawa, ON K1H 8L1, Canada; jbrophy@cheo.on.ca; 3 Department of Pediatrics, University of Ottawa, Ottawa, ON K1H 8M5, Canada; 4 Centre de recherche Azrieli du CHU Sainte Justine, Montréal, QC H3T 1C5, Canada; 5 Département de Microbiologie, Infectiologie et Immunologie, Université de Montréal, Montréal, QC H3T 1J4, Canada; 6 Division of Infectious Diseases, Hospital for Sick Children, Department of Pediatrics, University of Toronto, Toronto, ON M5G 1X8, Canada; 7 Department of Pediatrics, Alberta Children’s Hospital, University of Calgary, Calgary, AB T3B 6A8, Canada

**Keywords:** newborn screening, congenital cytomegalovirus, dried saliva spot screening

## Abstract

The purpose of this research was to evaluate the implementation and relative diagnostic accuracy of dried blood spot (DBS) and dried saliva spot (DSS)-based newborn screening for congenital cytomegalovirus infection (cCMV). This prospective paired-sample pilot studied consecutive births at 10 centers using DBS and DSS specimens from 12 February 2024 to 31 May 2025, using laboratory-developed qPCR assays. Newborns who screened positive on either specimen were offered urine qPCR confirmatory testing. Among 45,597 births, 39,047 newborns (86%) had satisfactory results in both specimens. Of the 150 newborns who screened positive, 39 underwent confirmatory urine testing within 21 days of life, and 24 had cCMV. As a percentage of DBS sensitivity, DSS screening displayed a non-significant relative sensitivity (rSe) of 109% [95CI: 97–123%]. The DSS relative false positive rate was 700% [95CI: 175–2795%] the DBS rate. Each newborn with cCMV identified by DSS but not DBS was associated with 6 additional false positives [95CI: 1.32–27.3]. Calculating rSe including newborns with urine CMV qPCR confirmation after day 21 showed DSS to be 128% [95CI: 111–147%] more sensitive than DBS. In this pilot of DSS screening for cCMV, DSS did not significantly increase sensitivity for cCMV and had an increased false positive rate. Screening with DSS extended turnaround times. Often, DSS screening for cCMV was not achieved within timeframes necessary to offer newborns a definitively diagnostic test to confirm congenital infection. The performance profile of DSS-based screening did not support population-wide implementation of DSS in our program.

## 1. Introduction

As recognition of congenital cytomegalovirus infection (cCMV) as an important pediatric health concern has grown, advocacy for universal newborn screening (NBS) to detect this infection has similarly expanded [1]. CMV is the most common congenital infection, the most common cause of non-genetic sensorineural hearing loss (SNHL) in childhood, and is responsible for a significant burden of neurodevelopmental disability [2,3]. Meta-analyses estimate that infection is prevalent in 0.48% of live births in developed countries [4] and only 10–15% of infections are clinically apparent [5]. Antiviral treatment is recommended for newborns with moderate to severe cCMV disease, many of whom may go unrecognized in the absence of a universal screening program [6].

NBS programs that have implemented population-based cCMV screening of newborns have thus far used CMV polymerase chain reaction (PCR) assays of dried blood spot (DBS) samples collected shortly after birth. The sensitivity and specificity of DBS screening (84% and 99%, respectively) [7] has been reported to be lower than that of saliva-based screening (99%, 100%) [8]. These systematic reviews show a remarkably wide range of observed sensitivity in DBS-based screening (28–100%), underscoring the need for an explanation of this inconsistency from further study of cCMV screening tests. Saliva testing is more convenient and more acceptable as a new initial sample than urine and shedding in saliva is similar to shedding in urine and considerably higher than shedding of virus in blood.

Since 2019, the province of Ontario has offered universal DBS-based NBS for cCMV. Formally, the programmatic target of this screen in Ontario is cCMV and the target of identification is childhood-onset SNHL. The screen-detected birth prevalence of cCMV in this province has been lower than expected (0.13%) [9] based on reports of cCMV in comparable populations [10]. Although a lower true rate of cCMV cannot be excluded, incomplete detection of affected newborns with DBS-based screening is also possible. We evaluated cCMV screening using dried saliva spots (DSS) collected in parallel to the DBS to: (i) assess whether a DSS-based screening strategy had greater sensitivity for cCMV compared to the existing DBS-based strategy; and (ii) determine whether the performance characteristics of a DSS-based test could increase the diagnostic yield of cCMV screening. This report describes the implementation and relative programmatic accuracy of a DSS-based cCMV compared to a DBS-based cCMV screening strategy. Diagnostic qPCR-based detection of CMV DNA in urine sampled ≤ 21 days of life served as the reference standard.

## 2. Materials and Methods

### 2.1. Participants

Live-born neonates at ten Ontario birthing hospitals between 12 February 2024, and 31 May 2025, were consecutively eligible, with up to 11-week deferments of start date permitted to accommodate site-specific implementation logistics. Eligible families resided in Ontario, and newborns could not be receiving palliative care at the time of screening. Beginning 1 March 2024, guardian consent for cCMV screening was obtained as part of the combined viral and genetic risk factor screen for permanent hearing loss we have previously described [9,11]. After sample collection, in-laboratory specimen receipt, and consent acquisition, cCMV assays were requested from the lab after specimens and consent had been electronically matched and linked. Explicit consent for screening was waived during the first study month under a temporary pandemic-related policy. Non-participation was identified by linking screening records with a provincial birth registry. The institutional research ethics board determined informed consent was not required to report this pilot quality improvement initiative. This report conforms to STARD standards [12].

### 2.2. Blood and Saliva Specimens

Recommendations instructed simultaneous collection of DBS and DSS specimens at 24 h of age. Blood and saliva were applied to separate Whatman 903 filter-paper. Limited newborn and maternal demographics and collection details were recorded by hospital staff on an attached form. Blood collection instructions specified heel lancing followed by direct spotting of blood to filter paper. Syringe- and tube-assisted blood collection was also accepted. Saliva was collected by gently rubbing a Puritan Hydroflock swab between the cheek pouches and gums then rolling the swab to saturate two filter paper spots. Repeated swabbing was advised if insufficient saliva was obtained. Saliva collection was to occur a minimum of one hour after the most recent breastmilk feed to minimize contamination with breast milk.

### 2.3. Initial and Confirmatory Screening Assays

Our laboratory methods were previously described [9]. Briefly, laboratory-developed qPCR assays were used to assess all specimens collected prior to the 21st day of life that had suitable quantity and quality. Primers and probes for two regions of the CMV genome (UL55 and UL83) were multiplexed with primers and probes for T-Cell Receptor Excision Circles (TREC) as part of newborn screening for severe combined immune deficiency, and RNase P RNA Component H1 (*RPP1*–HGNC:19273) as an internal control for DNA quantity as the initial assay. Any samples which had a *RPP1* Ct ≤ 30 cycles in DBS, ≤38 cycles in DSS or with any detectable CMV signal (Ct > 0) advanced to a confirmatory assay. The confirmatory assay assessed *UL55*, *UL83* and *RPP1* in duplicate with a method with more input DNA, higher reaction volume, and unchanged cycle thresholds. Insufficient *RPP1* at the confirmatory stage (same cutoffs as initial assay) was deemed unsatisfactory. Any samples with CMV signal in the confirmatory assay were evaluated by an accredited molecular scientist to classify as screen positive, screen negative, or unsatisfactory while unblinded to the newborn’s results in any other specimen. Seven months after the study commenced, our laboratory qPCR instruments were replaced and a new master mix was introduced. Extensive validations confirmed this change did not impact CMV sensitivity prior to implementation. Newborns without results from both specimen types due to specimen non-submission or unsatisfactory tests were excluded from accuracy calculations.

### 2.4. Diagnostic Reference Standard

In newborns, qPCR detection of CMV DNA in urine collected at ≤21 days of life is considered a sensitive diagnostic biomarker of cCMV [13]. Urine qPCR was offered to newborns screening positive on either specimen as well as their twins. Urine qPCR testing was ordered for diagnosis in some cases by hospital clinicians when cCMV was clinically suspected prior to the availability of screening results. Laboratories transmitted the date of urine collection and CMV qPCR result to a centralized provincial database. Only the single earliest urine test was considered in newborns repeatedly tested. Newborns who did not have urine testing (‘lost to follow-up’) and the urine results of newborns only tested due to a positive screen in their twin were excluded from accuracy calculations.

### 2.5. Clinical Follow-Up of Newborns

Follow-up of newborns with screen positive results was provided by clinicians unblinded to specimen-specific screening results. Diagnostic standards and Ontario’s classification system for cCMV disease have been previously detailed [9]. Newborns underwent an audiologist-administered hearing assessment, physical examination, head ultrasound, bloodwork (complete blood count, alanine aminotransferase, bilirubin) and urine CMV qPCR; newborns with one or more symptoms were also referred for ophthalmologic exam. Audiologist-identified hearing loss is reported directly to the Newborn Screening Ontario (NSO) by the provincial Infant Hearing Program.

### 2.6. Principal Analysis

Newborn characteristics are described within the pilot sample and overall population. Screening flow and timing are reported with medians and quartiles of age. We report relative measures of diagnostic accuracy on the multiplicative scale [14] because the reference standard test was only extended to newborns who screened positive in this study. Consistent with international guidelines, cCMV status in accuracy calculations only considered urine results sampled ≤ 21 days of life [15,16,17]. Relative sensitivity (rSe) (i.e., DSS sensitivity divided by DBS sensitivity) and relative false positive rate (rFPR) (i.e., DSS false positive rate divided by DBS false positive rate) were calculated with 95% confidence intervals [18]. The false positive to true positive ratio (FP:TP) is a measure of the false positive cost of increased yield added by a more sensitive but less specific test. The ratio conveys the number of uninfected newborns that a DSS-based strategy would forward to follow-up in order to identify a single infected newborn missed by DBS-based screening alone [19].

### 2.7. Sample Size

Sample size was calculated for a one-way superiority analysis of rSe. We conservatively aimed to identify an rSe of 1.10 with 95% confidence at 80% power. The probability of a newborn with cCMV but screening DBS-negative and DSS-positive was input as 16%, and the probability of the inverse discordancy was assumed to be less than 1%. This required 140 newborns with cCMV to receive DSS, DBS, and urine qPCR [20]. Assuming a 0.45% birth prevalence and incorporating a 10% rate of expected attrition, recruitment targeted at least 34,354 newborns.

### 2.8. Secondary Analysis of Diagnostic Accuracy

Measures of diagnostic accuracy may vary according to the definition of disease used to classify diagnostic outcomes in studies of screening tests [21]. The intended and primary analysis of our data evaluated only the infants who had confirmatory CMV testing prior to 21 days. The rSe and rFPR was recalculated for a secondary analysis using a definition of cCMV that includes any CMV detection in a confirmatory specimen cCMV regardless of the newborn’s age at the time the confirmatory specimen was sampled [22,23].

## 3. Results

### 3.1. Study Population

**Figure 1:**
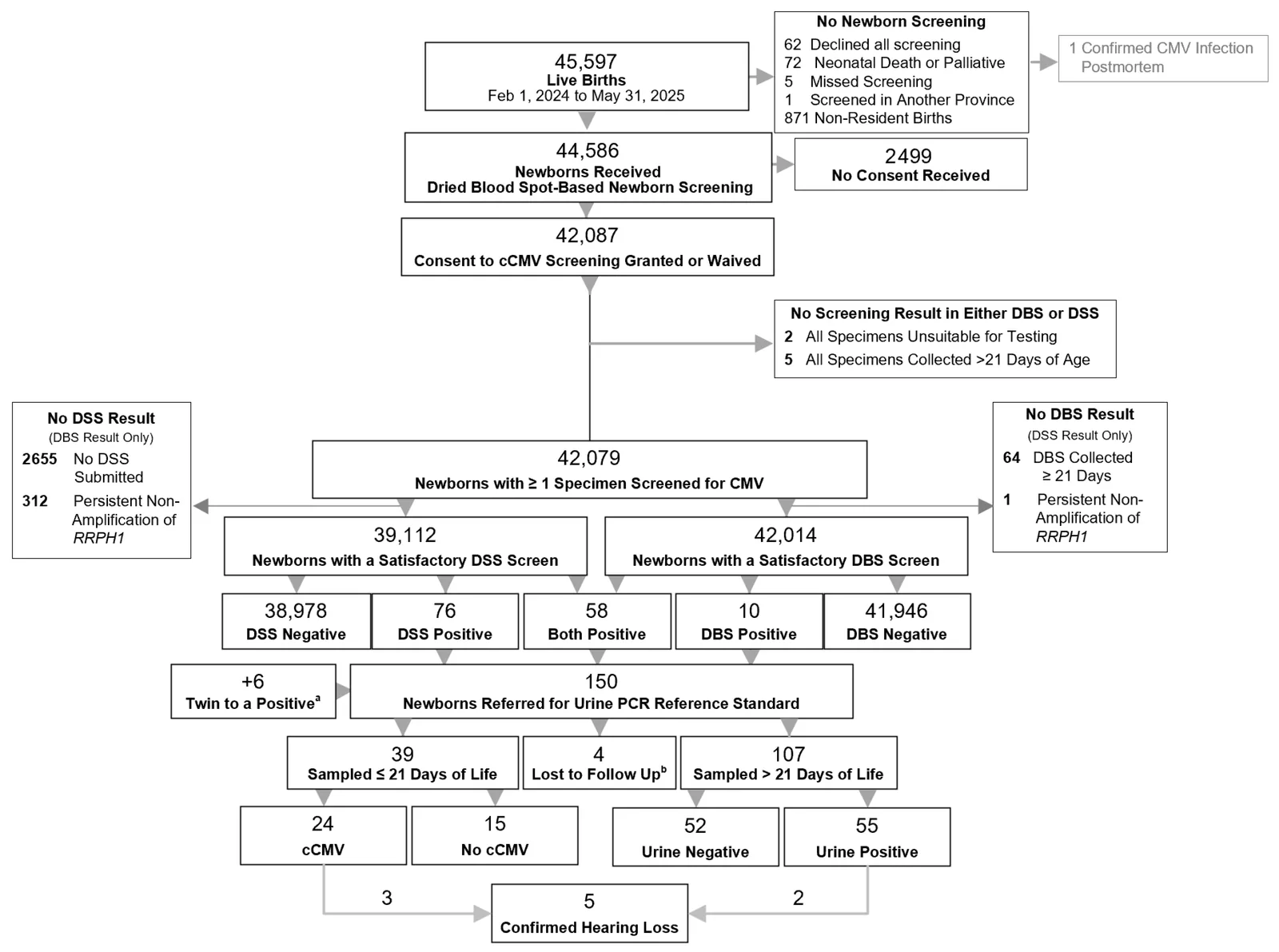
Screening Flow. ^a^ All twins were screen-negative by DBS. Five were also negative by DSS and one was unsatisfactory on DSS. ^b^ One newborn lost to follow-up was screen-positive by both DBS and DSS. All others were DSS screen-positive, DBS screen-negative.

**Figure 2:**
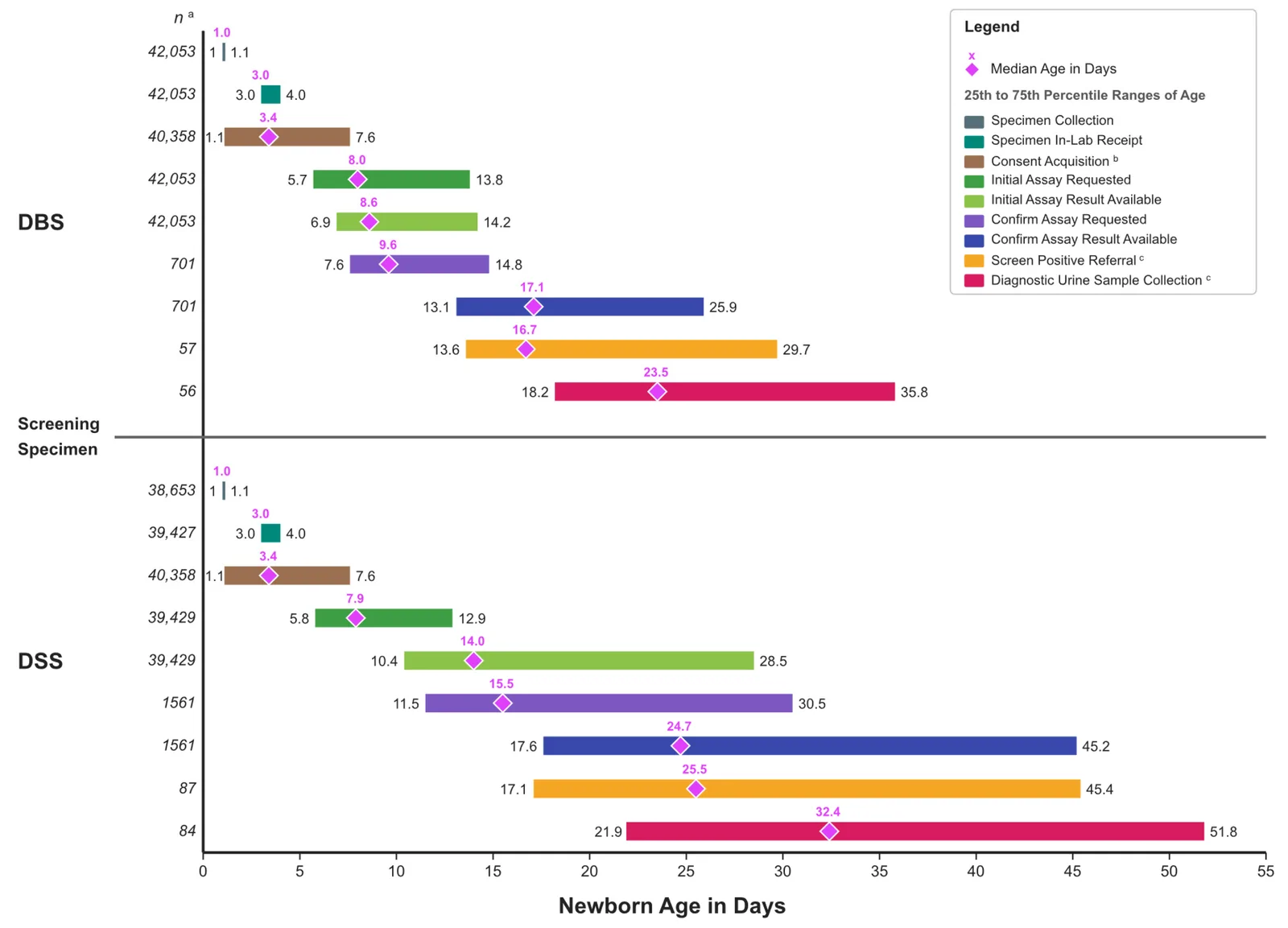
Timeliness of Screening and Diagnostic Testing. ^a^ Newborns with missing or unknown dates of collection, consent, or diagnostic urine sample collection were excluded. ^b^ Excludes 967 newborns for whom consent was waived under a pandemic-related policy. ^c^ Newborns positive on DSS and DBS are shown only once based on the chronologically earliest specimen to screen positive.

The consent rate among guardians of eligible newborns who received NBS was 94.3% (Figure 1). No reports of newborns with negative screens being diagnosed with cCMV clinically (false negatives) were received by NSO; 0.0% and 6.3% of infants did not have a DBS or DSS sample, respectively. Relative to newborns born elsewhere in the Province of Ontario, infants born at the 10 participating centres were slightly more likely to be male, to not be breastfed, and to be born to older mothers (Table 1). Newborn ages at specimen sample collection [median: 1 day, 25th–75th: 1.0–1.1] and subsequent receipt in-lab [3.0, 1.1–7.6] were identical across specimen types. The DSS specimens became more delayed than DBS at the availability of initial assay results due to batching of samples in the laboratory and delays persisted at subsequent time points (Figure 2).

### 3.2. Laboratory Analysis

Non-amplification of *RPP1* caused significantly fewer unsatisfactory results in DBS (0.03%) than DSS (1.16%, *p* < 0.0001). Retests resolved initial non-amplification in 14 of 15 DBS samples. In 68% of initially non-amplifying DSS samples, non-amplification of the internal control persisted across re-attempts, preventing a screening result. This implied an inadequate saliva sample in the DSS. Another contributor to analytical delays impacting DSS results was a contamination event that required a pause of DSS assays to allow for decontamination of the laboratory facilities. This event may have been due to dust created in punching very high-titre saliva samples and is a potential drawback of this sample type. Lastly, the lower volume of received DSS specimens relative to the provincial volume of DBS resulted in DSS assays pending longer than DBS assays prior to complete allocation of all wells.

### 3.3. Diagnostics and Follow-Up

Overall, 150 newborns were referred for confirmatory urine qPCR, of whom 144 had positive screening results (Table 2). Six newborns who screened negative were referred jointly with their positive twin. All five infants with hearing loss were positive by DBS. One-third (8/24) of newborns with cCMV confirmed by urine qPCR at ≤21 days had been diagnosed based on clinical suspicion prior to the availability of screening results.

### 3.4. Screening Strategy Performance

Across both diagnostic age groupings, the highest positive predictive values (PPV) for confirmed cCMV were observed when both DBS and DSS specimens screened positive (Table 2). In the primary analysis of our screening performance, where infants confirmed within 21 days of life were evaluated, there was no gain in sensitivity using DSS (Table 3). However, in a secondary analysis when positive CMV qPCR of urine collected at any age was attributed to cCMV, the DSS-based screen was significantly more sensitive than DBS.

## 4. Discussion

In this prospective paired-sample pilot evaluation embedded within a high-volume newborn screening laboratory, we found no statistically significant evidence that the sensitivity of a DSS-based cCMV screening strategy exceeded that of a DBS-based screening strategy among newborns undergoing diagnostic urine tests within 21 days of life. Adding DSS screening to a DBS-based screen could produce six additional false positive results for every additional newborn with cCMV in excess of screening with DBS alone. The increased false positive rate was also clinically impactful during our pilot. At the request of medical teams receiving the referrals, follow-up care delivery was modified for newborns with DSS-positive results who were negative by DBS screening. These infants only proceeded to comprehensive evaluation after a positive urine test. Newborns with DBS-positive results proceeded directly to full workup throughout the study. Audiologic surveillance of screen-positive newborns will continue until age five to detect late-onset hearing loss, but it is reassuring that all newborns with congenital-onset hearing loss in this pilot were identified through DBS-based screening alone. The increased burden of initial investigations required for newborns ultimately determined to be falsely positive together with the limited additional yield led to a decision not to implement DSS-based screening population-wide in Ontario. Previous literature on infants with CMV detected in saliva due to breastmilk contamination demonstrated low saliva sample viral loads that allowed discrimination from infants with true cCMV; we expected to be able to determine a qPCR-based threshold for true versus contaminated saliva samples; however, this was not the case.

The potential for the use of dried saliva samples in CMV screening was described over a decade ago. Boppana and colleagues found high sensitivity and specificity using the methodology [23]. Recently, the Minnesota screening program and our program have embarked on large trial tests of this method of detection. The observed 700% rFPR with the DSS-based strategy aligns with Minnesota’s recent comparison of a dried saliva swab-based cCMV test to two DBS-based methods, which found saliva generated false positive results at an 8–10x greater rate than DBS [24]. Among NBS programs, false positive results are tolerated to maximize sensitivity, with permissiveness generally calibrated to the severity and lethality of the screened condition. Our screening results among newborns without hearing loss at birth also reflect those of Minnesota, where salivary screening identified only three additional newborns with cCMV disease across seven years of study. Together these large-scale screening studies suggest that saliva-based cCMV screening may be unable to materially increase diagnostic yield for hearing loss.

An approach to cCMV screening using wet saliva samples rather than saliva dried on filter paper has been considered. Saliva samples were found to have good agreement with urine samples in a study of 2816 infants in Brazil [25]; however, saliva samples are not standard for newborn screening programs and would present a logistical challenge. A pooled saliva approach, followed by urine studies in infants that were members of the pool was later evaluated in a two-hospital study [26]. This approach is intriguing because it is low cost but may be difficult on a state, national or provincial scale where transfer of samples and discharge would interfere with the required rapid acquisition of confirmatory urine specimens.

It has been proposed that prospective population-based pilots are required prior to the adoption of candidate universal screening tests [27]. This design best informs the expected performance of tests implemented at mass scale in newborn populations. We submit evidence that, outside the strict controls offered by research study settings, DSS specimens were less likely to provide an actionable cCMV screening result than DBS. If a DBS-based screen had not been offered in this pilot, 7% of newborns would have gone unscreened. The high rate of non-submission of DSS specimens was the primary contributor to this attrition, which may suggest greater difficulty in obtaining salivary samples at the point of care relative to blood.

An important limitation of our study is the lack of oral swabs collected into liquid medium as the gold standard sample and hence the unknown sensitivity of DSS. This may also show a need for expanded training on neonatal salivary sampling, although detailed training materials were provided to all centres in this study. A second factor was the higher proportion of DSS samples that persistently failed to amplify human DNA. Feedback from staff collecting samples at sites was that babies’ mouths were often dry at the 24 h collection time while awaiting mothers’ breastmilk supply to come in; this could have contributed to the low DNA (both human and CMV) content found in DSS. The required re-testing increased laboratory workload and contributed to a delay in the availability of DSS screening results. Our challenges in attaining complete population coverage with DSS-based tests support the conclusion that DBS specimens likely enable the broadest possible access to cCMV screening.

Another important limitation to our work is that a minority of participants in this study who were referred due to DSS were able to complete confirmatory urine testing prior to 21 days of age. This interval is important because CMV infection is widely prevalent in the maternal population, readily transmitted postnatally to newborns, and with increasing neonatal infection it becomes impossible to determine whether CMV is congenitally acquired. Urine testing prior to 21 days has been considered standard for confirmation of cCMV status in accuracy calculations [15,16,17]. As mentioned above, when we re-evaluated our data including urine results from all participants, regardless of when urine sampling occurred, DSS had a significantly increased relative sensitivity (1.28-fold DBS sensitivity) and the ratio of false positives to true positives declined. However, we interpret these results with caution because confirmatory tests performed after 21 days may reflect postnatal transmission of CMV. In Canada, 13% of infants shed CMV DNA in urine by the third month of life [28]. The risk of misclassification led to the exclusion of most urine results from our principal analysis.

Lastly, we also recognize that molecular scientists and providers of follow-up care were not blinded to specimen-specific screening results. Although this reflects real-world laboratory review practices and information routinely provided to clinicians providing follow-up care, bias may have been introduced. Our study did not administer the reference urine test to a population-based subset of screen negatives, precluding the calculation of absolute sensitivity and specificity. Relative measures of diagnostic accuracy remained possible to calculate and better informed our comparative question.

## 5. Conclusions

We report the second multi-centre newborn screening pilot comparing a dried saliva-based cCMV to a DBS-based cCMV screening strategy. Our study was unable to precisely quantify the relative sensitivity of DSS-based screening in comparison to DBS; however, DSS screening did generate false positive results at a meaningfully higher rate. DSS, as collected in our study, fit imperfectly into Ontario’s DBS-based newborn screening workflow typical of NBSPs in North America, with analytical issues including repeated failed tests contributing to increased staff workload and extended result delays. These delays extended the screening process beyond 21 days of age, preventing most newborns with screen-positive DSS results from accessing a diagnostic test capable of definitively confirming their result. The certainty and scale of downstream false positive impacts paired with an uncertain sensitivity benefit did not support the implementation of screening for cCMV with DSS in Ontario.

## Figures and Tables

**Table 1:** Newborn and Specimen Characteristics.

	Study Sample	Provincial Births ^a^
	n (%)	n (%)
Newborns	42,087 (100)	140,673 (100)
Sex		
Male	21,989 (52.2)	72,184 (51.3)
Female	20,094 (47.7)	68,486 (48.7)
Screening Result and Basis		
cCMV Screen-Positive and Twin Siblings	150 (0.4)	175 (0.1)
DBS and DSS Tested	147	2 ^b^
Only DBS Tested	3	173
cCMV Screen-Negative	41,939 (99.6)	140,275 (99.7)
DBS and DSS Tested	38,900	349 ^b^
Only DBS Tested	2964	139,926
Only DSS Tested	65	
No cCMV Screening Result	7 (0.0)	223 (0.2)
Neither DBS or DSS Tested	7	223
Breastfeeding		
Exclusive Breastfeeding	20,595 (48.9)	72,279 (51.4)
Mixed Breastfeeding	15,160 (36.0)	47,801 (34.0)
Non-Breastfeeding	6305 (15.0)	16,531 (11.8)
Maternal Age in Years		
<25	2351 (5.6)	10,023 (7.1)
25–29	8061 (19.2)	33,687 (23.9)
30–34	16,742 (39.8)	57,351 (40.8)
≥35	14,334 (34.1)	37,855 (26.9)

^a^ Births in the study period excluding those at designated hospital centers. ^b^ Newborns sampled for DSS at a participating centre despite birth occurring elsewhere were excluded from this study (ex. inbound transfers of care).

**Table 2:** Reference Standard Results by Screening Result Combination and Age.

Newborn Group		Screening Result Combination ^a^		CMV DNA in Urine		
	n	DBS	DSS	Detected n (%) ^b^	Not Detected n (%)	Not Tested n (%)
All Screened Newborns	38,905	Negative	Negative	-	5 (0.0)	38,900 (100)
	8	Positive	Negative	3 (37.5)	5 (62.5)	-
	76	Negative	Positive	19 (25.0)	54 (71.1)	3 (3.9)
	58	Positive	Positive	55 (94.8)	2 (3.4)	1 (1.7)
	2	Positive	Not Determined	2 (100)	-	-
	2965	Negative	Not Determined	-	1 (0.0)	2964 (100)
	65	Not Determined	Negative	-	-	65 (100)
	8	Not Determined	Not Determined	-	-	8 (100)
Screen Positives with Urine Sampled Within 21 Days of Life	1	Positive	Negative	-	1 (100)	-
	15	Negative	Positive	2 (13.3)	13 (86.6)	-
	23	Positive	Positive	22 (95.7)	1 (4.3)	-

^a^ Screening results were Not Determined when either (i) a specimen of that type was not received; or (ii) the specimen received was unsuitable for testing; or (iii) if upon testing the specimen did not amplify the assay’s internal control. ^b^ This is the Positive Predictive Value (PPV).

**Table 3:** Analysis of Screening Accuracy.

	Primary Analysis		Secondary Analysis	
Age Limit to Consider Urine CMV DNA Detection as Confirmatory of cCMV	≤21 Days of Life		No Age Limit	
Screen-Positive Newborns Included	39		138 ^a^	
Screening Accuracy Measure	Estimate	95 CI	Estimate	95 CI
Relative Sensitivity (rSe)	1.09	0.97–1.23	1.28	1.11–1.47
Relative False Positive Rate (rFPR)	7.00	1.75–27.95	8.00	3.74–17.11
FP:TP Ratio	6:1	1.32–27.28	3:1	1.60–5.88

**^a^** Excludes two newborns without a DSS screening result, six twins of screen positive newborns, and four newborns who were lost to follow-up.

## Data Availability

Disclosure of the dataset used in this study is governed by local law. Researchers seeking access may do so through the research disclosure process outlined in Section 44 of the Province of Ontario’s *Personal Health Information Protection Act*.

## Abbreviations

cCMV	Congenital Cytomegalovirus Infection
CMV	Cytomegalovirus
DBS	Dried Blood Spot
DNA	Deoxynucleic Acid
DSS	Dried Saliva Spot
FP:TP	Ratio of False Positives to True Positives
NBS	Newborn Screening
NBSP	Newborn Screening Program
pCMV	Postnatal Cytomegalovirus Infection
QPCR	Quantitative Polymerase Chain Reaction
PHL-RF	Combined Risk Factor Screening of Risk Factors for Permanent Hearing Loss
PPV	Positive Predictive Value
RPP1	Human Ribonuclease P RNA Component H1
SNHL	Sensorineural Hearing Loss
STARD	Standards for Reporting Diagnostic Accuracy
TREC	T-Cell Receptor Excision Circles
UL55	Human Cytomegalovirus Envelope Glycoprotein B Gene (gB)
UL83	Human Cytomegalovirus Tegument Phosphoprotein pp65 Gene

## References

[1] Sánchez S.M., Kettler M., Basuray R.G., Malhotra P.S., Sanchez P.J. (2025). The Case for Universal Newborn Congenital Cytomegalovirus Screening. Clin. Perinatol..

[2] Goderis J., De Leenheer E., Smets K., Van Hoecke H., Keymeulen A., Dhooge I. (2014). Hearing Loss and Congenital CMV Infection: A Systematic Review. Pediatrics.

[3] Swanson M.R., Haisley L.D., Dobyns W.B., Schleiss M.R. (2025). Beyond hearing loss: Exploring neurological and neurodevelopmental sequelae in asymptomatic congenital cytomegalovirus infection. Pediatr. Res..

[4] Ssentongo P., Hehnly C., Birungi P., Roach M.A., Spady J., Fronterre C., Wang M., Murray-Kolb L.E., Al-Shaar L., Chinchilli V.M. (2021). Congenital Cytomegalovirus Infection Burden and Epidemiologic Risk Factors in Countries With Universal Screening: A Systematic Review and Meta-analysis. JAMA Netw. Open.

[5] Dollard S.C., Grosse S.D., Ross D.S. (2007). New estimates of the prevalence of neurological and sensory sequelae and mortality associated with congenital cytomegalovirus infection. Rev. Med. Virol..

[6] Sorichetti B., Goshen O., Pauwels J., Kozak F.K., Tilley P., Krajden M., Gantt S. (2016). Symptomatic Congenital Cytomegalovirus Infection Is Underdiagnosed in British Columbia. J. Pediatr..

[7] Wang L., Xu X., Zhang H., Qian J., Zhu J. (2015). Dried blood spots PCR assays to screen congenital cytomegalovirus infection: A meta-analysis. Virol. J..

[8] Rady S.M.A., Abdelmoati M.I., Sabra S., Alameddine M., Dsilva S., Shetty J.K. (2025). Diagnostic Performance of Salivary PCR for the Detection of Congenital Cytomegalovirus: A Systematic Review and Meta-Analysis. Viruses.

[9] Dunn J.K.E., Chakraborty P., Reuvers E., Gallagher L., Kernohan K.D., Lacaria M., Barton M., Leifso K., Pernica J.M., Santander E. (2025). Outcomes of a Population-Based Congenital Cytomegalovirus Screening Program. JAMA Pediatr..

[10] Dollard S.C., Dreon M., Hernandez-Alvarado N., Amin M.M., Wong P., Lanzieri T.M., Osterholm E.A., Sidebottom A., Rosendahl S., McCann M.T. (2021). Sensitivity of Dried Blood Spot Testing for Detection of Congenital Cytomegalovirus Infection. JAMA Pediatr..

[11] Kernohan K.D., Gallagher L., Pigeon M., Yeh E., Lacaria M., Axford M.M., MacCormick J., Papaioannou V., Quercia N., Rupar C. (2025). Newborn screening for common genetic variants associated with permanent hearing loss: Implementation in Ontario and review of the first 3 years. Genet. Med..

[12] Bossuyt P.M., Reitsma J.B., Bruns D.E., Gatsonis C.A., Glasziou P.P., Irwig L., Lijmer J.G., Moher D., Rennie D., de Vet H.C.W. (2015). STARD 2015: An Updated List of Essential Items for Reporting Diagnostic Accuracy Studies. Clin. Chem..

[13] Lanzieri T.M., Caviness A.C., Williams J.J., Demmler-Harrison G. (2025). Cytomegalovirus urinary excretion in children with congenital and postnatally acquired infection. J. Clin. Virol..

[14] Schatzkin A., Connor R.J., Taylor P.R., Bunnag B. (1987). Comparing New And Old Screening Tests When A Reference Procedure Cannot Be Performed On All Screenees. Am. J. Epidemiol..

[15] Barton M., Forrester A.M., McDonald J. (2020). Update on congenital cytomegalovirus infection: Prenatal prevention, newborn diagnosis, and management. Paediatr. Child Health.

[16] Council of State and Territorial Epidemiologists 23-ID-02 Standardized Surveillance Case Definitions for Congenital Cytomegalovirus (cCMV) Infection and Disease. https://cdn.ymaws.com/www.cste.org/resource/resmgr/ps/ps_2023/23-ID-02_cCMV.pdf.

[17] Leruez-Ville M., Chatzakis C., Lilleri D., Blazquez-Gamero D., Alarcon A., Bourgon N., Foulon I., Fourgeaud J., Gonce A., Jones C.E. (2024). Consensus recommendation for prenatal, neonatal and postnatal management of congenital cytomegalovirus infection from the European congenital infection initiative (ECCI). Lancet Reg. Health-Eur..

[18] Pepe M.S. (2001). Comparing disease screening tests when true disease status is ascertained only for screen positives. Biostatistics.

[19] Chock C., Irwig L., Berry G., Glasziou P. (1997). Comparing dichotomous screening tests when individuals negative on both tests are not verified. J. Clin. Epidemiol..

[20] Pepe M.S. (2004). The Statistical Evaluation of Medical Tests for Classification and Prediction.

[21] Newman T.B., Kohn M.A. (2020). Critical Appraisal of Studies of Diagnostic Test Accuracy. Evidence-Based Diagnosis: An Introduction to Clinical Epidemiology.

[22] Boppana S.B., Ross S.A., Novak Z., Shimamura M., Tolan R.W., Palmer A.L., Ahmed A., Michaels M.G., Sánchez P.J., Bernstein D.I. (2010). Dried blood spot real-time polymerase chain reaction assays to screen newborns for congenital cytomegalovirus infection. JAMA.

[23] Boppana S.B., Ross S.A., Shimamura M., Palmer A.L., Ahmed A., Michaels M.G., Sánchez P.J., Bernstein D.I., Tolan R.W.J., Novak Z. (2011). Saliva polymerase-chain-reaction assay for cytomegalovirus screening in newborns. N. Engl. J. Med..

[24] Schleiss M.R., Osterholm E.A., Shanley R., Hernandez-Alvarado N., Graupmann E., Kruc R., Jansen R., Polonenko M., Herd H., Fernandez-Alarcon C. (2025). Dried Blood Spot PCR for Detection of Congenital Cytomegalovirus Infection and Disease. JAMA Netw. Open.

[25] Yamamoto A.Y., Mussi-Pinhata M.M., Marin L.J., Brito R.M., Oliveira P.F.C., Coelho T.B. (2006). Is saliva as reliable as urine for detection of cytomegalovirus DNA for neonatal screening of congenital CMV infection?. J. Clin. Virol..

[26] Merav L., Ofek Shlomai N., Oiknine-Djian E., Caplan O., Livneh A., Sido T., Peri A., Shtoyer A., Amir E., Ben Meir K. (2024). Implementation of pooled saliva tests for universal screening of cCMV infection. Nat. Med..

[27] Calonge (2022). Ned Chair Letter to cCMVNominators to, R.USP. https://www.hrsa.gov/sites/default/files/hrsa/advisory-committees/heritable-disorders/rusp/chair-letter-ccmv-nominators.pdf.

[28] Sbihi H., Simmons K.E., Sears M.R., Moraes T.J., Becker A.B., Mandhane P.J., Subbarao P., Dai D.L.Y., Finlay B.B., Turvey S.E. (2022). Early-life cytomegalovirus infection is associated with gut microbiota perturbations and increased risk of atopy. Pediatr. Allergy Immunol..

